# Peroxiredoxin 1 Promotes Proinflammatory Cytokine Secretion in Human Dysplastic Oral Keratinocytes and Mouse Tongue Precancerous Tissues

**DOI:** 10.1155/ancp/6577043

**Published:** 2025-03-30

**Authors:** Min Zhang, Wenjing Li, Jing Li, Wenchao Wang, Lingyu Li, Yunping Lu, Min Wang, Xiaofei Tang

**Affiliations:** ^1^Beijing Institute of Dental Research, Beijing Stomatological Hospital and School of Stomatology, Capital Medical University, Fengtai District, Beijing 100070, China; ^2^Department of Pathology, Beijing Stomatological Hospital and School of Stomatology, Capital Medical University, Fengtai District, Beijing 100070, China; ^3^Department of Prosthodontics, Beijing Stomatological Hospital and School of Stomatology, Capital Medical University, Fengtai District, Beijing 100070, China

**Keywords:** inflammatory factors, NF-κB, oral leukoplakia, Prx1

## Abstract

Oxidative stress, a widespread phenomenon involved in many pathological conditions, may be closely related with the progression of oral leukoplakia (OLK). Under chronic inflammation, oxidative stress could stimulate the local formation of a tumor-specific microenvironment in some types of cancer, though not well defined in oral cancer even in OLK. Peroxiredoxin 1 (Prx1), a widely expressed sulfhydryl antioxidant protein, is overexpressed in various tumors and affects tumorigenesis, cell proliferation, apoptosis, invasion, and metastasis. Prx1 also acts as a potent proinflammatory factor. Nuclear factor (NF)-κB is a member of the core dimeric transcription factor family that coordinates the inflammatory response. To investigate the role of Prx1 in oxidative stress-related inflammation in OLK, a coculture model of human dysplastic oral keratinocyte (DOK) and human epidermal fibroblast (HFF) was established and stimulated with H_2_O_2_. Cellular reactive oxygen species (ROS) and Prx1 levels in DOK were determined via flow cytometry and western blotting, respectively. Additionally, the levels of the inflammatory factors, interleukin (IL)-6, IL-8, IL-10, and interferon (IFN)-γ, in the conditioned medium were determined via enzyme-linked immunosorbent assay (ELISA). DOK nuclear expression of NF-κB was detected via immunofluorescence assay and western blotting. Moreover, the expression levels of inflammatory factors and the nuclear expression of NF-κB were examined in 4-nitroquinoline-1-oxide (4NQO)-induced tongue precancerous tissues of mice. H_2_O_2_ increased Prx1 levels and nuclear expression levels of NF-κB in DOKs and mouse tongue precancerous tissues and elevated the levels of IL-6, IL-8, IL-10, and IFN-γ secreted in the culture supernatants and mouse tongue tissues. However, *Prx1* knockdown in DOK and mouse tongue tissues attenuated the upregulation of inflammatory factor and nuclear NF-κB expression levels. Overall, our results suggest that oxidative stress increases Prx1 expression, which promotes inflammatory factor expression by activating NF-κB in human DOK and mouse tongue precancerous tissues.

## 1. Introduction

Oral leukoplakia (OLK) is a potentially malignant disorder, with ~0.13%–34% of OLK cases progressing to squamous cell carcinoma [1, 2]. Oxidative stress is one of the main risk factors for OLK development and progression, and prolonged oxidative stress may lead to chronic inflammation [3]. Under chronic inflammation, oxidative stress causes various types of damage to nucleic acids, proteins, and lipids, resulting in tissue damage [4]. It is reported that these inflammation-related damages could stimulate a local tumor-specific microenvironment in lesions and may play critical roles in some tumor genesis [5]. However, it is not so well defined in oral cancer, and the specific correlation between oxidative stress-induced inflammation and OLK progression remains to be elucidated.

Peroxiredoxin 1 (Prx1), a sulfhydryl antioxidant protein, participates in oxidative stress; directly interacts with various transcription factors, including c-Myc and nuclear factor (NF)-κB, in the nucleus; and is involved in biological regulatory processes. Prx1 is overexpressed in various tumors, including lung, bladder, liver, thyroid, and breast cancers, and affects tumorigenesis, cell proliferation, apoptosis, invasion, and metastasis [6–8]. Prx1 is significantly overexpressed in human oral squamous cell carcinoma (OSCC) and oral mucosa OLK, and its expression increases with oxidative damage during the progression from simple to abnormal hyperplasia in cancerous lesions [9, 10]. Therefore, oxidative stress-related OLK is closely associated with increased Prx1 expression, indicating Prx1 as a potential marker of OLK malignancy. Prx1 acts as a potent proinflammatory factor [11]. However, the mechanism by which Prx1 regulates oxidative stress-induced inflammation in OLK remains unknown. Therefore, in this study, we investigated the roles of Prx1 in regulating oxidative stress-related inflammation in OLK and explored the underlying mechanisms using in vitro cultured precancerous dysplastic oral keratinocytes (DOKs) and wild-type and *Prx1*-knocked down tongue precancerous lesion mouse models.

## 2. Materials and Methods

### 2.1. Cell Culture and Prx1 Knockdown in DOKs

DOKs (European Collection of Authenticated Cell Cultures [ECACC] 94122104) were kindly donated by Dr. Qianming Chen (Zhejiang University, China) and authenticated by Beijing Microread Genetics Co., Ltd. (Beijing, China), with a 100% match for the ECACC human cell line(s), DOK, in the ECACC short tandem repeat (STR) database. The cells were maintained in a high-glucose Dulbecco's Modified Eagle's medium (Gibco, USA) supplemented with 10% fetal bovine serum (Gibco) and 5 µg/mL hydrocortisone (Beyotime, China) in a 37°C humidified incubator with 5% CO_2_. shPrx1 (5′-CTCTTGACTTCACCTTTGTGT-3′) or negative control (NC) were transfected into DOKs, and the stable *Prx1*-knocked down cells were selected using 1 µg/mL puromycin (Sigma, USA) for 1 week.

### 2.2. Establishment of a Coculture Model With Precancerous DOKs and Human Epidermal Fibroblasts (HFFs)

A HFF–DOK coculture model was established to simulate the local microenvironment of OLK. Transwell plates were inserted into each well of a 24-well plate, and HFFs (3 × 10^5^ cells/well) were seeded in the 24-well plate. After 2 h, 8 × 10^3^ DOKs were plated in the upper chamber of the Transwell. Cells were treated with 3% H_2_O_2_ in a culture medium containing 2% Ultroser G (Serum substitute; Saibaiao, China) for 3 and 6 h. The culture supernatant was collected, cell fragments were removed via centrifugation, and the conditioned medium was stored at −80°C.

### 2.3. Reactive Oxygen Species (ROS) Assay

DOKs (1 × 10^6^) were seeded in a 6-cm dish for 24 h and treated with the conditioned medium for 6 h. Cells were collected and stained with 10 μM 2′,7′-dichlorodihydrofluorescein diacetate (DCFH-DA; Beyotime) for 20 min at 37°C. A BD Accuri C6 flow cytometer (BD Bioscience, USA) was used to measure the intracellular ROS levels. DOKs (1 × 10^5^) were seeded in a 24-well plate for 24 h and treated with the conditioned medium for 6 h. Cells were stained with 10 μM DCFH-DA at 37°C for 20 min. ROS accumulation in the cells was observed under a fluorescence microscope (IX71; OLYMPUS, Japan).

### 2.4. Enzyme-Linked Immunosorbent Assay (ELISA)

Interleukin (IL)-6, IL-8, IL-10, and interferon (IFN)-γ Human ELISA kits (Thermo Fisher Scientific, USA) were used in the study. Assays were performed in three independent experiments, following the manufacturer's instructions. Briefly, all reagents in the ELISA kit and samples were brought to room temperature, according to the manufacturer's instructions. After calculating the number of wells required for each sample, the enzyme label strip was removed from the aluminum foil bag. The plate was washed thrice with the 1× washing buffer at 300 µL/well. Standards and samples were added to the plate at 100 µL/well and incubated at room temperature for 2 h. The liquid was discarded, and the plate was washed thrice with the 1× washing buffer at 300 µL/well. The working solution of antibody was added to the enzyme label plate at 100 µL/well and incubated at room temperature for 1 h. After washing thrice with the 1× washing buffer, 200 µL/well of the substrate working solution was added to the enzyme label plate, mixed, and incubated for 20 min at room temperature out of direct sunlight. Then, 50 µL/well termination solution was added with gentle shaking until even color development was achieved. Finally, absorbance was measured at 450 nm using a Multi-Mode Detection Platform (Spectra Max Paradigm, USA).

### 2.5. Immunofluorescence Assay

DOKs were plated at a density of 1 × 10^5^ cells/well in a 24-well plate, incubated in a 37°C incubator with 5% CO_2_ overnight, and treated with the conditioned medium for 6 h. DOKs were fixed with ice-cold 100% ethanol for 15 min, washed with phosphate-buffered saline (PBS), punctured with 0.5% Triton for 15 min, blocked with 1% bovine serum albumin for 30 min, and incubated overnight with the rabbit anti-NF-κB p65 antibody (1:500; Abcam, USA). The cells were washed with PBS and incubated with the goat antirabbit IgG antibody (1:500; CST). Nuclei were stained with 4′, 6-diamidino-2-phenylindole solution (10 µg/mL) for 5 min, and the fluorescence signal was measured using the fluorescence microscope (IX71; OLYMPUS).

### 2.6. Western Blotting

Total protein and nuclear protein were extracted from DOKs, and protein concentration was measured using the Bradford method. Equal amounts of protein samples were separated on a 10% gel via sodium dodecyl sulfate-polyacrylamide gel electrophoresis, transferred to a polyvinylidene difluoride membrane, and blocked with 5% skim milk for 1 h. After incubation with the rabbit anti-Prx1 primary antibody (1:5000; Abcam) and the rabbit anti-NF-κB p65 antibody (1:1000; CST) overnight at 4°C, the membrane was incubated with the appropriate secondary antibody for 1 h and analyzed using an enhanced chemiluminescence reagent (BioRad, USA).

### 2.7. Ethics Statement and Animal Studies

All animal experiments adhered to the National Research Council's Guide for the Care and Use of Laboratory Animals and were approved by the Animal Ethics and Welfare Committee of Beijing Stomatological Hospital (approval no. KQYY-202103-005, Beijing, China). Briefly, a 4-nitroquinoline-1-oxide (4NQO)-induced tongue carcinogenesis model was established using wild-type (Prx1+/+, C57BL/6) and *Prx1* knockdown (Prx1+/−, C57BL/6) mice (age: 6–8 weeks old; weight: 25–30 g), as previously described [12]. The mice were randomly divided into six groups: wild-type control (*n* = 6, half male and half female), wild-type 4NQO (*n* = 12, half male and half female), wild-type 4NQO + H_2_O_2_ (*n* = 12, half male and half female), *Prx1* knockdown blank (*n* = 6, half male and half female), *Prx1* knockdown 4NQO (*n* = 12, half male and half female), and *Prx1* knockdown 4NQO + H_2_O_2_ (*n* = 12, half male and half female) groups. The mice in experimental groups were first provided water containing 50 μg/mL 4NQO every day for 16 weeks and then distilled water for 2 weeks, whereas the mice in control groups were provided distilled water only. As for 4NQO + H_2_O_2_ treatment groups, the mice were provided 50 μg/mL 4NQO every day and coated with 3% H_2_O_2_ on the tongue mucosa three times a week for 16 weeks and then distilled water for 2 weeks. All mice were euthanized via intraperitoneal injection of sodium pentobarbital (250 mg/kg) after 18 weeks of treatment. Their tongues were cut out and dissected longitudinally, half of them were frozen at −80°C for quantitative reverse transcription-polymerase chain reaction (qRT-PCR), and the other half were fixed with formalin for 24 h for hematoxylin and eosin (H&E) and immunohistochemical staining.

### 2.8. H&E Staining

Tongue tissues were cut into 4-μM-thick sections. After H&E staining, the sections were examined under an Olympus BX61 microscope (OLYMPUS). Based on the categorization of head and neck tumors by the World Health Organization (5th Edition, 2022), histological alterations in the tongue mucosa and severity of lesions were noted by two pathologists.

### 2.9. Immunohistochemistry

For the dewaxing and rehydration of tissue sections, antigen retrieval was carried out by heating the sections for 10 min in a pressure cooker containing 10 mM citrate buffer (pH 6.0) for the detection of IL-6, IL-8, IL-10, IFN-, 8-hydroxydeoxyguanosine (8-OHdG), and Prx1, whereas the ethylenediaminetetraacetic acid repair procedure was used to detect NF-B and Ki67. Endogenous peroxidases were neutralized with 3% H_2_O_2_. After blocking, the sections were incubated with antibodies against IL-6 (1:200; ABclonal, China), IL-8 (1:200; ABclonal), IL-10 (1:200; ABclonal), IFN-γ (1:200; Bioss, China), 8-OHdG (1:200; Abcam), Prx1 (1:500; Abcam), and NF-κB (1:800; CST, USA), and Ki67 working solution (Maxim Biotechnologies, China) overnight at 4°C, followed by incubation with secondary antibodies for 30 min at room temperature. Freshly produced 3,3′-diaminobenzidine solution (Maxim Biotechnologies) was used for staining, and the nuclei were counterstained with hematoxylin. For NC, PBS was used as substitute for primary antibodies and the same for other procedures. In each slice, three fields were selected for image acquisition using an Olympus BX61 microscope. To investigate the correlation between Prx1 and the inflammatory response in OLK, in 4NQO and 4NQO + H_2_O_2_ group, the tongue mucosa of five mice with the severity of the majority of lesions from each group was randomly selected and included in the correlation statistics of immunohistochemical staining. The Image-Pro Plus program (Media Cybernetics, MD, USA) was used for quantitative analysis. Cells positive for Ki67 and NF-κB immunohistochemical staining were counted, and the mean optical density (MOD) was calculated as follows: MOD = integrated optical density (IOD)/measured area.

### 2.10. qRT-PCR

TRIZOL (Thermo Fisher Scientific, USA) was used to extract the total RNA from mouse tongue tissues, and the cDNA Reverse Transcription Kit (CoWin Biosciences, China) was used to reverse-transcribe 2 μg RNA to cDNA. qRT-PCR was carried out using the SYBR Green PCR Master Mix (CoWin Biosciences) and 1 μL aliquots of cDNA as templates. Sangon Biotech (Shanghai, China) designed all the primers listed in Table 1.

### 2.11. Statistical Analyses

IBM SPSS v.20.0 software was used to analyze the results. Data are expressed as the mean ± standard deviation. The homogeneity of variance and normal distribution were tested. One-way analysis of variance and *t*-test were used for comparisons, and statistical significance was set at *p* < 0.05.

## 3. Results

### 3.1. H_2_O_2_ Induces Cellular ROS Accumulation in DOKs

**Figure 1:**
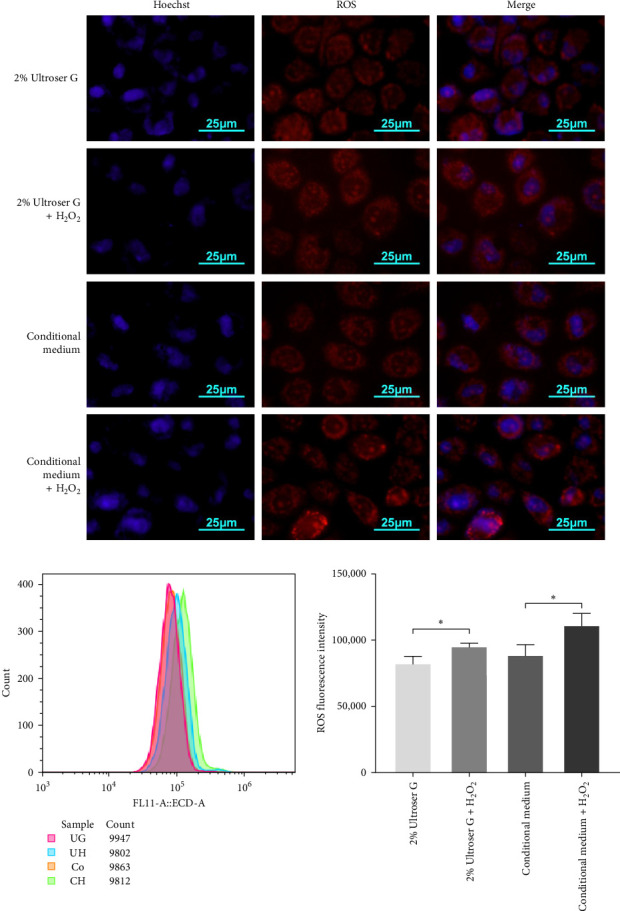
H_2_O_2_ increases the reactive oxygen species (ROS) levels in dysplastic oral keratinocytes (DOKs) in the human epidermal fibroblast (HFF)–DOK coculture and control media. (a) Fluorescence microscopy display of cellular ROS levels in DOKs induced by 0.3 mM H_2_O_2_ for 6 h. (b) Flow cytometric analysis of cellular ROS levels in DOKs induced by 0.3 mM H_2_O_2_ for 6 h. Bar graph indicates the ROS fluorescence intensity. Data are represented as the mean ± standard deviation (SD). *⁣*^*∗*^*p* < 0.05.

A coculture model of DOKs and HFFs was established and exposed to H_2_O_2_ to simulate the local microenvironment of OLK. Here, cells were treated with 0.3 mM H_2_O_2_ for 6 h to induce oxidative stress. DOKs were cultured in a conditioned medium prepared from the supernatant of the coculture system and fluorescently stained with dihydroethidium. Cellular ROS levels were determined via fluorescence microscopy and flow cytometry. As shown in Figure 1a, 0.3 mM H_2_O_2_ treatment enhanced the red fluorescence intensity in DOKs in both 2% Ultroser G and conditioned medium. Compared to the intensity in DOKs cultured in 2% Ultroser G, the intracellular red fluorescence intensity was slightly enhanced in the conditioned medium-cultured DOKs under the same H_2_O_2_ treatment conditions.

Fluorescence intensity was also quantitatively measured using flow cytometry, and the results are shown in Figure 1b. Intracellular ROS levels increased after H_2_O_2_ treatment in both DOKs cultured in the conditioned medium and those cultured in 2% Ultroser G (*p* < 0.05). ROS levels in DOKs cultured in the conditioned medium were slightly higher than those in the DOKs cultured in 2% Ultroser G; however, the difference was not statistically significant. Overall, 0.3 mM H_2_O_2_ was found to induce oxidative stress in DOKs.

### 3.2. H_2_O_2_ Increases the Cellular Prx1 Expression Levels in DOKs

**Figure 2:**
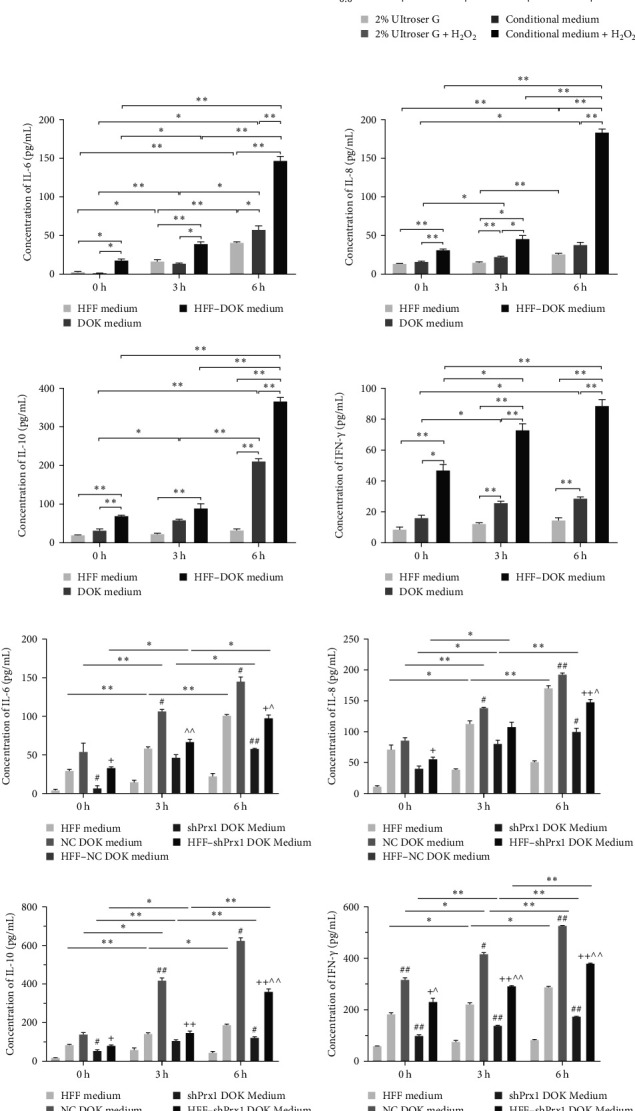
H_2_O_2_ upregulates the expression of peroxiredoxin 1 (Prx1) in DOKs in the coculture medium, and *Prx1* knockdown decreases the secretion of interleukin (IL)-6, IL-8, IL-10, and interferon (IFN)-γ in the HFF–DOK coculture supernatant induced by H_2_O_2_. (a) Western blotting revealed the expression of Prx1 in DOKs induced by 0.3 mM H_2_O_2_ for 6 h. β-Actin served as a loading control. Bar graph shows the gray scale value. (b) Enzyme-linked immunosorbent assay (ELISA) revealed the IL-6, IL-8, IL-10, and IFN-γ levels in the HFF–DOK coculture, HFF, and DOK supernatants. (c) ELISA revealed the IL-6, IL-8, IL-10, and IFN-γ levels in HFFs, DOKs, shPrx1 DOKs, HFF–DOK coculture, and HFF–shPrx1 DOK coculture supernatant. Data are represented as the mean ± SD. *⁣*^*∗*^*p* < 0.05 and *⁣*^*∗∗*^*p* < 0.01; #*p* < 0.05 and ##*p* < 0.01 compared with the NC control; ^*p* < 0.05 and ^^*p* < 0.01 compared with the HFF-NC; +*p* < 0.05 and ++*p* < 0.01 compared with the shPrx1 group.

DOKs were treated with 0.3 mM H_2_O_2_ for 6 h under various culture conditions. In Figure 2a, western blotting revealed that Prx1 levels were significantly increased in the DOKs cultured in both 2% Ultroser G and conditioned medium (*p* < 0.05 and *p* < 0.01, respectively) after 0.3 mM H_2_O_2_ treatment for 6 h; however, no significant differences were observed between 2% Ultroser G and conditional medium. These results suggest that H_2_O_2_-induced oxidative stress increases Prx1 expression in DOKs.

### 3.3. H_2_O_2_ Promotes the Secretion of IL-6, IL-8, IL-10, and IFN-γ in Culture Supernatants

HFFs, DOKs, and HFF–DOK coculture models were treated with 0.3 mM H_2_O_2_ for 3 and 6 h, and the conditioned medium was collected. The levels of inflammatory factors were determined using ELISA. As shown in Figure 2b, IL-6, IL-8, IL-10, and IFN-γ levels were the highest in the coculture conditioned medium (*p* < 0.01) and the lowest in the HFF medium, and the inflammatory factor levels increased in a time-dependent manner with H_2_O_2_ treatment. Prx1 levels in DOKs and inflammatory factor levels in the medium were upregulated in a time-dependent manner with H_2_O_2_ treatment. However, whether Prx1 is related to the inflammatory response of DOKs remains unclear.

### 3.4. Prx1 Knockdown Attenuates the H_2_O_2_-Induced Increase in Inflammatory Factor Levels in Culture Supernatants

To further explore the roles of Prx1 in the inflammatory response of DOKs, stable *Prx1*-knocked down DOKs were established (Figure S1). In Figure 2c, ELISA revealed that the levels of IL-6, IL-8, IL-10, and IFN-γ in the coculture medium were increased in a time-dependent manner with H_2_O_2_ treatment. The levels of inflammatory factors were the highest in the HFF-NC DOK coculture medium, but they significantly reduced after *Prx1* knockdown in DOKs, suggesting that oxidative stress-induced Prx1 expression in DOKs positively regulates the inflammatory factor secretion in the culture medium.

### 3.5. Prx1 Knockdown Inhibits Tongue Epithelial Dysplasia in 4NQO-Induced Mouse Tongue Tissues

**Figure 3:**
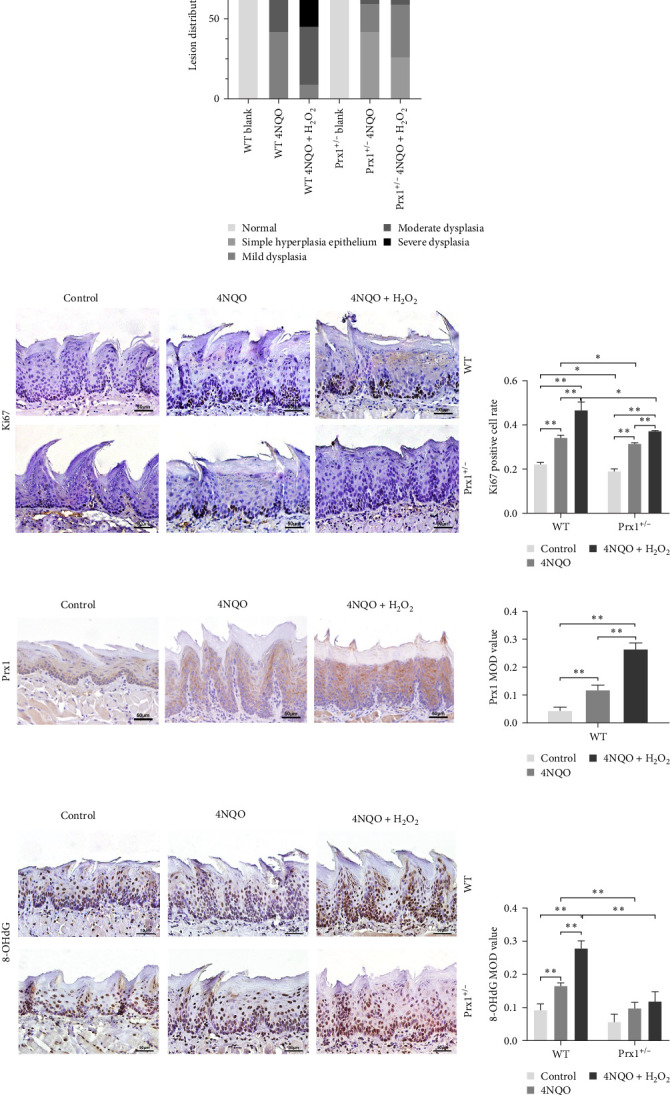
*Prx1* knockdown inhibits tongue epithelial dysplasia and oxidative DNA damage in 4-nitroquinoline-1-oxide (4NQO)-induced mouse tongue tissues. (a) Degree of lesions in mouse tongue tissues. (b) Ki67 staining of mouse tongue tissues in each group. (c) Immunohistochemical (IHC) staining for Prx1 in the mouse tongue premalignant lesions of wild-type mice. (d) Immunohistochemical staining for 8-hydroxydeoxyguanosine (8-OHdG) in the mouse tongue tissues of each group. Positive immunohistochemical staining images were analyzed using the Image-Pro Plus v.6.0 software, and the results are shown as bar graphs. *⁣*^*∗*^*p* < 0.05 and *⁣*^*∗∗*^*p* < 0.01 (200× magnification).

After 18 weeks of treatment, the tongue mucosa of normal control mice was soft and pink with a smooth surface, whereas that of the 4NQO and 4NQO + H_2_O_2_ groups was white, grainy, and slightly tough, with plaques observed in some mice. H&E staining revealed that the tongue mucosal epithelium exhibited clear cell layers with normal morphology in normal control mice but showed simple hyperplasia and dysplasia (mild, moderate, and severe) similar to the pathological changes in human leukoplakia in the 4NQO and 4NQO + H_2_O_2_ groups (Figure S2). As shown in Table 2 and Figure 3a, in wild-type mice, dysplasia was more obvious in the 4NQO + H_2_O_2_ group than in the 4NQO group (*p* < 0.05), but no significant difference was observed in the tongue lesions of 4NQO + H_2_O_2_ and 4NQO groups (*p* > 0.05) in Prx1 ± mice. In 4NQO and 4NQO + H_2_O_2_ groups, the severity of tongue lesions was significantly attenuated in Prx1 ± mice compared to that in the wild-type mice (*p* < 0.05).

### 3.6. Prx1 Knockdown Inhibits Cell Proliferation and DNA Damage in Mouse Tongue Tissues

Immunohistochemical staining in Figure 3b showed that, in wild-type mice, the rates of Ki67 (a mitotic marker)-positive cells in the tongue mucosa tissues of 4NQO and 4NQO + H_2_O_2_ groups were significantly increased (*p* < 0.01) compared with that in the blank control mice and were the highest in the tongue tissue of 4NQO + H_2_O_2_ group (*p* < 0.01). Regardless of 4NQO or 4NQO + H_2_O_2_ treatment, the positive rate of Ki67 in mouse tongue tissue was significantly decreased after *Prx1* knockdown compared to that in wild-type mice (*p* < 0.05).

Figure 3c displayed that in wild-type mice, compared with the control group, 4NQO induced Prx1 expression in the mouse tongue mucosa (*p* < 0.01), and 4NQO + H_2_O_2_ treatment further increased the Prx1 expression in the tongue mucosa (*p* < 0.01). Therefore, oxidative stress increased Prx1 expression in tongue precancerous lesions.

Notably, 8-OHdG is a biomarker of oxidative DNA damage. The levels of 8-OHdG in tongue tissues were determined via immunohistochemical staining. As shown in Figure 3d, compared with those in the blank control group, 8-OHdG levels were significantly increased in the tongue mucosa tissues of the 4NQO and 4NQO + H_2_O_2_ groups in wild-type mice (*p* < 0.01) and were the highest in the 4NQO + H_2_O_2_ group (*p* < 0.01). Moreover, 8-OHdG levels in the tongue mucosa tissues of 4NQO and 4NQO + H_2_O_2_ groups were significantly decreased in Prx1 ± mice than in the wild-type mice (*p* < 0.01).

These results suggest that oxidative stress increases Prx1 expression, thereby promoting cell proliferation in oral precancerous tissues. However, *Prx1* knockdown attenuates DNA damage in the precancerous mouse tongue tissues.

### 3.7. Prx1 Knockdown Reduces the Expression Levels of Inflammatory Factors in Mouse Tongue Tissues

**Figure 4:**
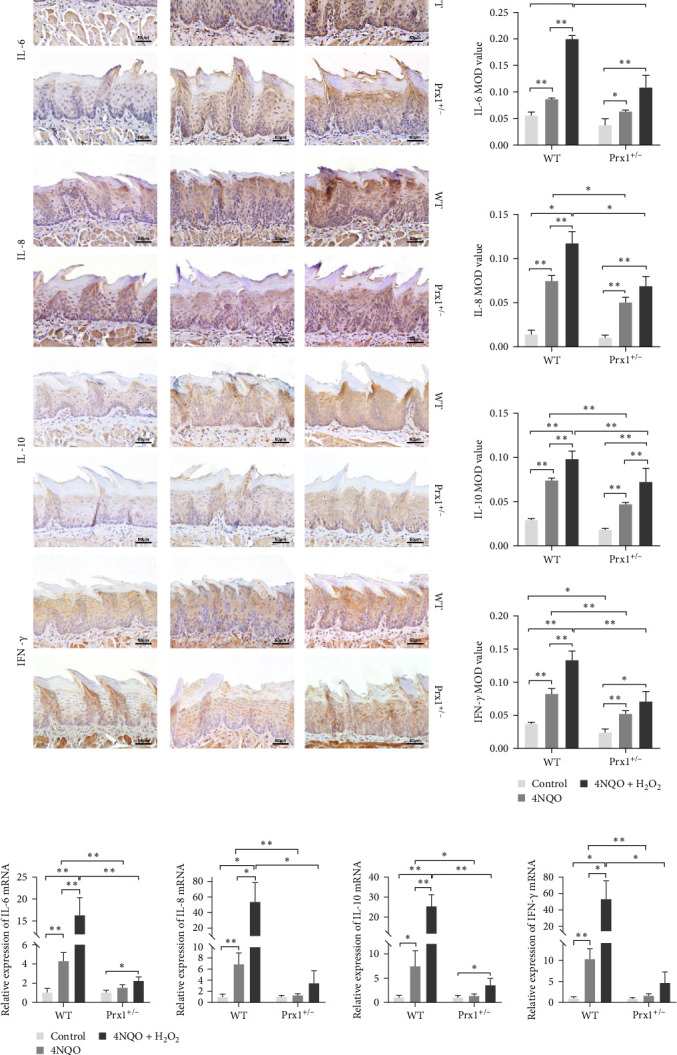
Expression levels of of IL-6, IL-8, IL-10, and IFN-γ are downregulated in the *Prx1*-knocked down mouse tongue tissues. (a) IHC staining for IL-6, IL-8, IL-10, and IFN-γ in the mouse tongue tissues (200× magnification). IHC images were analyzed using the Image-Pro Plus v.6.0 software, and the results are shown as bar graphs. *⁣*^*∗*^*p* < 0.05 and *⁣*^*∗∗*^*p* < 0.01. (b) mRNA expression levels of IL-6, IL-8, IL-10, and IFN-γ in mouse tongue tissues were determined via quantitative reverse transcription-polymerase chain reaction (qRT-PCR). *⁣*^*∗*^*p* < 0.05 and *⁣*^*∗∗*^*p* < 0.01.

Next, expression levels of inflammatory factors in mouse tongue tissues were measured using immunohistochemical staining. As shown in Figure 4a, in wild-type mice, the expression levels of IL-6, IL-8, IL-10, and IFN-γ were significantly upregulated in the 4NQO group than in the normal control and further increased in the 4NQO + H_2_O_2_ group with statistical significance (*p* < 0.01). Compared with those in the wild-type mice, IL-6, IL-8, IL-10, and IFN-γ levels in the tongue tissues of Prx1 ± mice were significantly decreased in the 4NQO and 4NQO + H_2_O_2_ groups (*p* < 0.01 and *p* < 0.05, respectively).

qRT-PCR was used to determine the mRNA levels of inflammatory factors in the tongue tissues of mice. As shown in Figure 4b, compared with those in the control group, mRNA levels of *IL-6*, *IL-8*, *IL-10*, and *IFN-γ* were upregulated significantly in the mouse tongue tissues of 4NQO group (*p* < 0.01 and *p* < 0.05, respectively) and further increased in the 4NQO + H_2_O_2_ group with statistical significance (*p* < 0.01 and *p* < 0.05, respectively). In Prx1 ± mice, mRNA levels of *IL-6* and *IL-10* in the tongue tissues of the 4NQO + H_2_O_2_ group were significantly higher than those in the tongue tissues of the blank control (*p* < 0.05). Compared with those in the wild-type mice, mRNA levels of *IL-6*, *IL-8*, *IL-10*, and *IFN-γ* in Prx1 ± mice were significantly decreased in both 4NQO and 4NQO + H_2_O_2_ groups (*p* < 0.01 and *p* < 0.05, respectively).

Oxidative stress increased the protein and mRNA expression levels of inflammatory factors, but these levels were decreased in Prx1 ± mice. Therefore, oxidative stress increases Prx1 expression, which positively regulates the secretion of inflammatory factors in precancerous lesions.

### 3.8. Prx1 Regulates the Inflammatory Factor Expression by Activating NF-κB in H_2_O_2_-Induced DOKs and 4NQO-Induced Mouse Tongue Tissues

**Figure 5:**
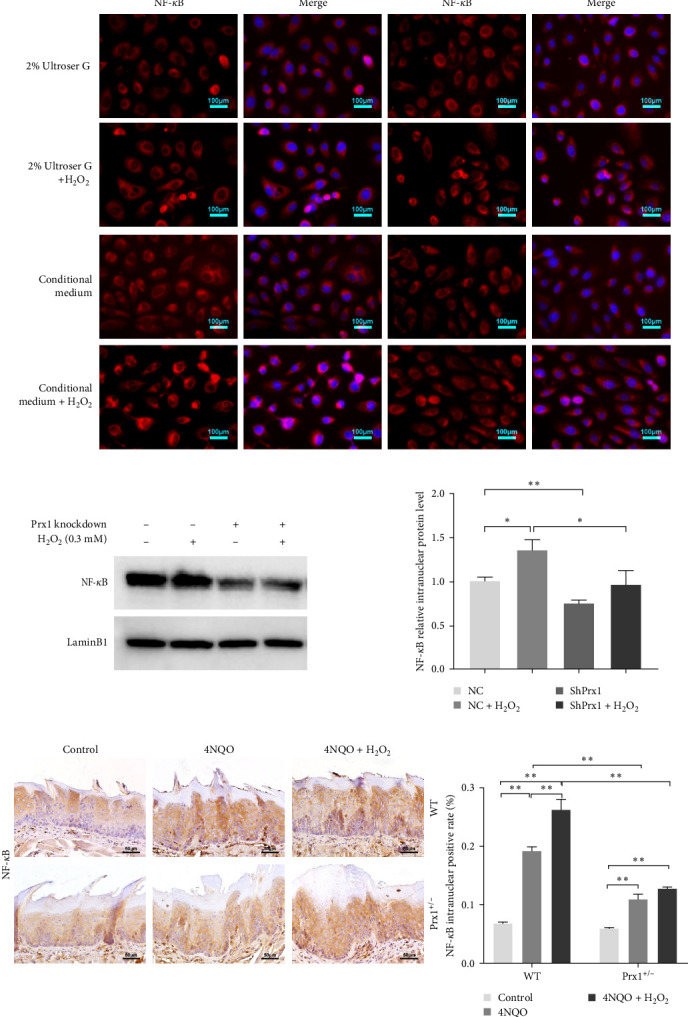
Prx1 regulates inflammatory factor expression by activating nuclear factor (NF)-κB in H_2_O_2_-induced DOKs and 4NQO-induced mouse tongue tissues under oxidative stress. (a) Immunofluorescent labeling of NF-κB in control and shPrx1 DOKs cultured in the HFF-NC DOK/shPrx1 DOK coculture medium. Cells were labeled with the anti-NF-κB p65 antibody (red), and nucleus were stained with 4′,6-diamidino-2-phenylindole (DAPI; blue). (b) Western blotting revealed the nuclear expression levels of NF-κB in the DOKs cultured in conditioned medium after 0.3 mM H_2_O_2_ treatment for 6 h. (c) Intranuclear expression of NF-κB in mouse tongue tissues was examined using IHC staining (200× magnification). Data are presented as mean ± SD, *⁣*^*∗*^*p* < 0.05, *⁣*^*∗∗*^*p* < 0.01.

The activation of the NF-κB signaling pathway is closely linked to the inflammatory response. Immunofluorescence assay showed that 0.3 mM H_2_O_2_ treatment for 6 h significantly increased the nuclear expression of NF-κB in DOKs. As shown in Figure 5a, red fluorescent dots of NF-κB in NC DOK nucleus increased significantly after H_2_O_2_ treatment. Compared with that in NC cells, the red fluorescent punctate of NF-κB was significantly reduced in the shPrx1 DOK nucleus. Under the same treatment with H_2_O_2_, accumulation of red fluorescent dots in the nucleus of DOKs cultured in the coculture conditioned medium increased significantly compared to that in those cultured in the control medium. As shown in Figure 5b, western blotting revealed that the nuclear expression levels of NF-κB were significantly increased in the DOKs cultured in conditioned medium (*p* < 0.05) after 0.3 mM H_2_O_2_ treatment for 6 h. Compared with that in NC cells and H_2_O_2_-induced NC cells, the nuclear expression levels of NF-κB were significantly reduced in the shPrx1 DOK under the same treatments (*p* < 0.01 and *p* < 0.05, respectively). Immunohistochemical staining was performed to determine the nuclear expression of NF-κB p65 in tongue mucosa tissues. Compared with that in the blank control mice, the expression levels of nuclear NF-κB were significantly increased in the tongue tissues of wild-type 4NQO and 4NQO + H_2_O_2_ mice (*p* < 0.01). Moreover, nuclear NF-κB levels were lower in 4NQO and 4NQO + H_2_O_2_ Prx1+/– mouse tongue mucosa tissues than in the wild-type tissues (*p* < 0.01; Figure 5c). *Prx1* knockdown reduced the nuclear expression of NF-κB in H_2_O_2_-induced DOKs and 4NQO-induced mouse tongue tissues and decreased the secretion of inflammatory factors, suggesting that Prx1 positively regulates the secretion of inflammatory factors by activating NF-κB in DOKs and 4NQO-induced mouse tongue tissues.

## 4. Discussion

Various inflammatory stimuli, such as excessive ROS/reactive nitrogen species produced during oxidative metabolism, trigger inflammatory responses and lead to the synthesis and secretion of proinflammatory cytokines. Prx1 is a member of the mercaptan-specific peroxidase family that is activated under oxidative stress and interacts with NF-κB, c-Myc, and androgen receptors to regulate various biological processes, such as cell differentiation, proliferation, and apoptosis [13]. Prx1 is overexpressed in many solid tumors [7, 14]. We previously reported increased expression of Prx1 in OLK tissues [9]. Prx1 is an effective proinflammatory factor, and tumor necrosis factor (TNF)-β, IL-1β, lipopolysaccharide, and TNF-α promote the release of Prx1 [15, 16]. As an intrinsic ligand of toll-like receptor-4, Prx1 promotes the expression of proinflammatory cytokines by activating NF-κB [17]. Prx1 also participates in the inflammatory response by binding to the macrophage migration inhibitory factor [11]. Here, Prx1 levels were increased in precancerous DOKs and 4NQO-induced mouse tongue precancerous tissues. Prx1 expression significantly increased with the dysplasia grade. These results suggest that oxidative stress-induced Prx1 expression promotes the development of oral precancerous lesions.

Under inflammatory conditions, inflammatory cells are recruited and secrete various factors that aggravate inflammation. IL-6, IL-8, IL-10, and IFN-γ are important inflammatory factors. IL-6 is a pleiotropic cytokine that plays important roles in the immune response, inflammation, and hematopoiesis. IL-6 is expressed in various tumor tissues, such as the breast, prostate, colorectal, and ovarian cancer tissues, and is involved in tumor cell apoptosis, growth, proliferation, migration, invasion, angiogenesis, and metastasis [18, 19]. IL-8 is a proinflammatory CXC chemokine that binds to two chemokine receptors, CXCR1 and CXCR2. IL-8 is produced by monocytes, endothelial cells, and various epithelial cells, and it induces granulocyte chemotaxis and phagocytosis [20]. Tumor-derived IL-8 activates endothelial cells in the tumor vasculature, promotes angiogenesis, and induces neutrophil chemotactic infiltration at the tumor site [21]. IL-10 was initially identified as a cytokine synthesis inhibitor because of its inhibitory effect on helper T cell cytokine production, which are produced by almost all leukocytes and many human tumor cells. In a mouse model of B16 melanoma, IL-10 promotes tumor growth by stimulating angiogenesis, tumor cell proliferation, and immunosuppression [22]. Incidence of skin cancer increases with IL-10 deficiency in a chemically induced mouse model, whereas IL-10 overexpression protects the mice from carcinogenesis [23]. IFN family comprises of multipotent cytokines with antiviral, antitumor, and immunomodulatory activities. As the central coordinator of immune response, IFN-γ affects the development, chemotaxis, and activation of immune cells and kills tumor cells by activating the immune response. IFN-γ can also promote tumor cell proliferation, tumor cell escape from cytotoxic T lymphocytes, and natural killer cell recognition and cytolysis [24].

Some scholars specifically investigated the roles of inflammatory mediators in the progression of OLK to OSCC and reported that IL-10 levels begin to increase in the early stages of leukoplakia. However, IFN-γ expression gradually decreases with the progression of lesions, suggesting that immunosuppression induced by chronic inflammation promotes the occurrence of OSCC [25]. Immunohistochemical detection of NF-κB and IL-6 in oral precancerous lesions and OSCC revealed that the staining intensity of NF-κΒ in cytoplasmic tissues increases gradually during transformation from normal mucosa to OSCC, with no significant correlation between NF-κΒ and IL-6; however, IL-6 participates in the local inflammatory response [26]. IL-8 expression levels in the tissues and saliva of patients with OSCC and precancerous lesions increase with the progression of lesions, suggesting IL-8 as a biomarker for the malignant transformation of oral mucosa [27]. In this study, oxidative stress significantly increased the levels of IL-6, IL-8, IL-10, and IFN-γ inflammatory factors in the DOK medium in a time-dependent manner. In the mouse model, the protein and mRNA levels of IL-6, IL-8, IL-10, and IFN-γ in the tongue precancerous lesions were also significantly increased via oxidative stress stimulation. These results suggest that oxidative stress promotes the progression of precancerous lesions and that local inflammation is aggravated with the development of lesions. The degree of epithelial dysplasia and expression of Prx1 in precancerous lesions are consistent with inflammation. DOKs and mouse models with stable *Prx1* knockdown were investigated to elucidate the mechanism by which Prx1 regulates the inflammatory response and affects the progression of precancerous lesions. We found that the expression of IL-6, IL-8, IL-10, and IFN-γ induced by oxidative stress was significantly reduced in the *Prx1*-knocked down DOKs and mouse tongue tissues than in the controls, suggesting that *Prx1* knockdown decreases the inflammatory factor expression in tongue precancerous lesions.

NF-κB signaling pathway is closely associated with inflammation and activation of NF-κB/activator protein-1, and TNF-α regulates inflammation in various chronic diseases [28]. Disruption of the IκκB–NF-κB axis in Prx1 (+) fibroblasts promotes the development of atopic dermatitis [29]. Classical activation of NF-κB is achieved by the degradation of IκB or release of NF-κB transcription factor dimers, such as p50 and p65 (RelA), facilitating the translocation of unmasked NF-κB from the cytoplasm to the nucleus, regulating target gene transcription, and activating the cellular adaptive response to inflammation. In acute inflammation, NF-κB terminates its transcriptional activity via the upregulation of IκB family, leading to the partial inactivation of NF-κB. However, under chronic inflammatory conditions, persistent activation leads to increased NF-κB activity. Here, nuclear expression levels of NF-κB were significantly decreased in both *Prx1*-knocked down DOKs and Prx1 ± mouse tongue tissues compared to those in the controls, indicating that Prx1 promotes the inflammatory response in OLK by activating NF-κB.

In conclusion, our results revealed that Prx1 regulates inflammation by activating NF-κB, thereby facilitating the malignant transformation of OLK.

## Figures and Tables

**Table 1:** Sequences of primers used in this study.

Gene	Primer	Sequence
*IL-6*	Forward	5′-TGTGCAATGGCAATTCTGAT-3′
	Reverse	5′-GGTACTCCAGAAGACCAGAGGA-3′
*IL-8*	Forward	5′-AAGAAACCACCGGAAGGAAC-3′
	Reverse	5′-ACTCCTTGGCAAAACTGCAC-3′
*IL-10*	Forward	5′-GCTCTTACTGACTGGCATGAG-3′
	Reverse	5′-CGCAGCTCTAGGAGCATGTG-3′
*IFN-γ*	Forward	5′-ACAGCAAGGCGAAAAAGGATG-3′
	Reverse	5′-TGGTGGACCACTCGGATGA-3′
*GAPDH*	Forward	5′-TCTCCACACCTATGGTGCAA-3′
	Reverse	5′-CAAGAAACAGGGGAGCTGAG-3′

**Table 2:** The pathological diagnosis results of tongue tissue in each group of mice.

Pathological diagnosis	Group					
	Wild-type control (*n* = 6)	Wild-type 4NQO (*n* = 12)	Wild-type 4NQO + H_2_O_2_ (*n* = 12)*⁣*^*∗*^	Prx1^+/−^ control (*n* = 6)	Prx1^+/−^ 4NQO (*n* = 12)*⁣*^*∗*^	Prx1^+/−^ 4NQO + H_2_O_2_ (*n* = 12)^#^
Normal	6	—	—	6	—	—
Simple hyperplasia epithelium	—	—	—	—	5	3
Mild dysplasia	—	5	1	—	2	4
Moderate dysplasia	—	6	4	—	5	4
Severe dysplasia	—	1	6	—	—	1

*Note:* One death in the wild-type 4NQO + H_2_O_2_ group.

*⁣* ^*∗*^Indicates a statistical difference compared to the wild-type 4NQO group, *p* < 0.05.

^#^Indicates statistically different from the wild-type 4NQO + H_2_O_2_ group, *p* < 0.05.

## Data Availability

The data that support the findings of this study are available on request from the corresponding author, Xiaofei Tang, upon reasonable request.

## Supporting Information

Supporting Information Figure S1: Construction of stable Prx1-knocked down DOKs. Figure S2: Histopathology of the tongue tissues in mice.
