# Supplementary material for: Peroxiredoxin 1 Promotes Proinflammatory Cytokine Secretion in Human Dysplastic Oral Keratinocytes and Mouse Tongue Precancerous Tissues
Source: Anal Cell Pathol (Amst). 2025 Mar 30;2025:6577043. doi: 10.1155/ancp/6577043 (PMC11972860; doi:10.1155/ancp/6577043)
Supplement: Supporting Information — Figure S1: Construction of stable Prx1-knocked down DOKs. Figure S2: Histopathology of the tongue tissues in mice. [file 6577043.f1.pdf]

Supplementary Material

1. To further explore the roles of Prdx1 in the inflammatory response of DOKs, stable Prdx1-knocked down DOKs were established.
As shown in SFig.1, western blotting revealed that the expression levels of Prdx1 were significantly decreased in shPrdx1 DOKs compared to those in NCs, indicating that Prdx1 was stably knocked down in DOKs.

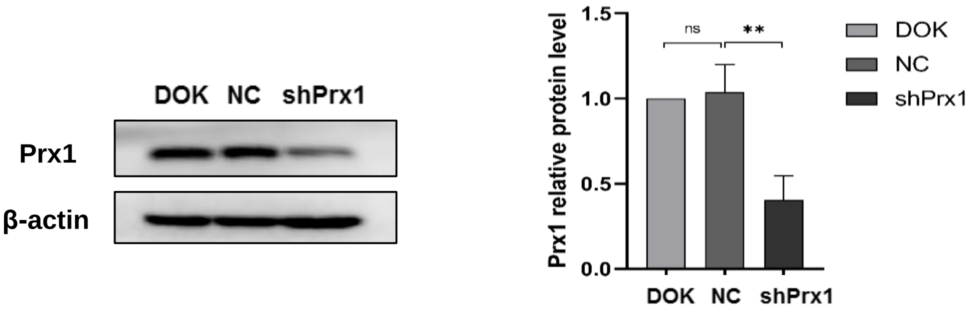

SFig.1 Construction of stable Prdx1-knocked down DOKs. Western blotting showed the expression levels of Prdx1 in shPrdx1 and control DOKs. β-actin served as a loading control. Data are represented as the mean ± SD. **P < 0.01.

2. After the 4NQO-induced mouse tongue precancerous lesion model was constructed, the degree of tongue mucosa lesions was determined under the microscope in each mouse, and normal mucosa, hyperplasia, mild dysplasia, moderate dysplasia and severe dysplasia could be observed (SFig.2).

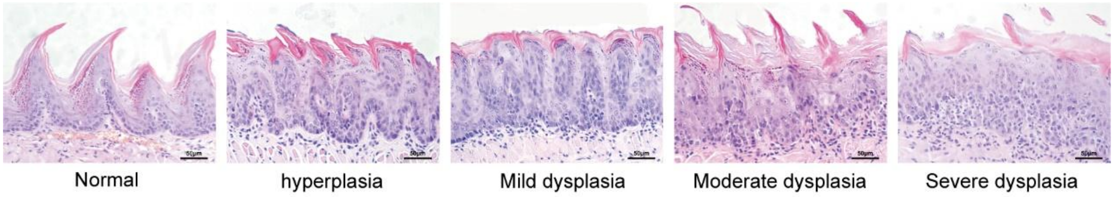

SFig.2 Histopathology of the tongue tissues in mice (magnification 200×)
